# Awareness and perceived fairness of option B+ in Malawi: a population-level perspective

**DOI:** 10.7448/IAS.20.1.21467

**Published:** 2017-03-08

**Authors:** Sara Yeatman, Jenny Trinitapoli

**Affiliations:** ^a^ Department of Health and Behavioral Sciences, University of Colorado Denver, Denver, CO, USA; ^b^ Department of Sociology, University of Chicago, Chicago, IL, USA

**Keywords:** HIV, ART, antiretroviral, Malawi, ethics, option B+

## Abstract

**Introduction**: Policies for rationing antiretroviral therapy (ART) have been subject to on-going ethical debates. Introduced in Malawi in 2011, Option B+ prioritized HIV-positive pregnant women for lifelong ART regardless of the underlying state of their immune system, shifting the logic of allocation away from medical eligibility. Despite the rapid expansion of this policy, we know little about how it has been understood and interpreted by the people it affects.

**Methods**: We assessed awareness and perceived fairness of the prioritization system for ART among a population-based sample of young women (n = 1440) and their partners (n = 574) in southern Malawi. We use a card-sort technique to elicit understandings of who gets ART under Option B+ and who should be prioritized, and we compare perceptions to actual ART policy using sequence analysis and optimal matching. We then use ordered logistic regression to identify the factors associated with policy awareness.

**Results**: In 2015, only 30.7% of women and 21.1% of male partners understood how ART was being distributed. There was widespread confusion around whether otherwise healthy HIV-positive pregnant women could access ART under Option B + . Nonetheless, more young adults thought that the fairest policy should prioritize such women than believed the actual policy did. Women who were older, more educated or had recently engaged with the health system through antenatal care or ART had more accurate understandings of Option B + . Among men, policy awareness was lower, and was patterned only by education.

**Conclusions**: Although most respondents were unaware that Option B+ afforded ART access to healthy-pregnant women, Malawians support the prioritization of pregnant women. Countries adopting Option B+ or other new ART policies such as universal test-and-treat should communicate the policies and their rationales to the public – such transparency would be more consistent with a fair and ethical process and could additionally serve to clarify confusion and enhance retention.​​

## Introduction

Since the introduction of antiretroviral therapy (ART), there have been vigorous debates over who should be prioritized for access to these lifesaving medicines. These debates have occurred in every context suffering an HIV epidemic but are most relevant in sub-Saharan Africa (SSA), which has the unfortunate distinction of being both the poorest of world regions and home to two-thirds of all HIV-positive people. This combination of characteristics means that there has always been, and continues to be, insufficient ART for all those in need.

Over the last decade, the context of ART in SSA has been revolutionized. What was once out of reach for poor and rural HIV-positive individuals has become widely available. Nonetheless, generalized epidemics are still contexts of tremendous demand for ART and limited supply, and policymakers continue to grapple with the ethical dilemmas associated with developing and revising systems of triage in a changing treatment landscape. Although the specifics of triage have evolved considerably [1–3], the general problems of scarcity and uneven access have been constant: where there is not yet universal access to ART, shifting policies organize the persistent reality that extending treatment to some means that others have to wait [4,5].

Consensus only recently solidified around the idea that starting ART early was beneficial for an individual’s health [6–10]. From 2002 until 2011, Malawi’s approach to distributing ART was consistent with the strategy pursued by most countries in the region: prioritize the sickest individuals for free ART [11,12]. Those with the most advanced disease – measured symptomatically with WHO staging criteria or CD4-count – received ART, but others whose immune systems had yet to deteriorate found their places on the ART registers only after having developed the same gruesome symptoms they witnessed in their neighbours.

In 2011, Malawi introduced a radical new policy called “Option B+” that provided lifelong access to ART to all HIV-positive pregnant or breastfeeding women regardless of the underlying state of their immune system [13,14]. The shift was meant to accomplish two goals: simplify the prevention of mother to child transmission (PMTCT) protocols and end the practice of frequently starting and stopping pregnant/breastfeeding women on ART for PMTCT purposes, which had become common in high fertility contexts and can exacerbate drug resistance [13,14]. The move to prioritize pregnant women through Option B+ was emblematic of a broader, region-wide interest in universal test-and-treat approaches [15] to enhance individual health [6–8], reduce onward transmission [16,17], and simplify initiation procedures [18]. Additionally, donors and policymakers have long been sympathetic to women, viewed as a particularly vulnerable group and the focus of a disproportionate share of HIV resources and programming [19–23].

Based on early indications of success in Malawi [24], the WHO recommended Option B+ as global policy in 2012 [25]; since then nineteen other African countries have adopted variants of the policy [26].

Throughout the rapid expansion of Option B+, there have been relatively few voices of caution or critique. Nonetheless, some have raised ethical concerns including the risk of impinging upon access for immuno-compromised men and non-pregnant women, the top-down nature of a policy developed with little-to-no input from communities, and the speed with which it was adopted and spread absent evidence of improved PMTCT outcomes [27–30].

Disagreement over the priority structure for rationing ART is inevitable [1,31,32], but there is ample guidance on what constitutes an ethical *process* of priority setting for the allocation of scarce health resources [4,33–36]. Daniels and Sabin’s [35] *accountability for reasonableness* approach to healthcare rationing argues that although experts will disagree about the best way to distribute ART, fair procedures are of tantamount importance, as the moral legitimacy requisite for rationing rests upon them. This requires priority-setting policies like Option B+ to be transparent, relevant to the populations affected, and open to revision [31,32,34]. Applied to the dilemma of ART scarcity in Malawi, transparency demands that the public be informed of both the prioritization structure underlying distribution and the rationale behind it. Relevance, on the other hand, refers to involving stakeholders (i.e. those most affected by policy) and implementing policies that reflect their principles.

In this paper, we explore the “unevenness” of access to ART [2] as perceived by young adults in southern Malawi. We assess understandings of the ART prioritization system under Option B+ and examine how the priorities of those living amidst an epidemic align with those developed in Geneva and Lilongwe.

## Methods

Our data come from Tsogolo la Thanzi (TLT), a longitudinal population-based study in Balaka, Malawi. The TLT-2015 sample followed women first interviewed in 2009, a refresher sample of women first interviewed in 2012, and the current male sexual partners of female respondents. The original TLT sample was drawn as a simple random sample of women aged 15–25 living within 7-kilometers of Balaka’s main market, and the 2012 refresher sample was drawn from the same sampling frame. Women were given tokens for their male partners who could then enrol themselves in the study [37]. Surveys were administered face-to-face in Chichewa by trained local interviewers in private rooms at a central research centre. Our full analytic sample consists of 1440 women and 574 male partners.

Prior to the 2015 survey, TLT conducted a brief audit of all the clinics in the baseline catchment area offering antenatal care services (*n *= 14). The vast majority of these clinics began to implement Option B+ in 2011 or 2012 (*n *= 12), but two in the most rural outskirts of the catchment area did not introduce the programme until 2013. By the time of the TLT-2015 survey, all antenatal-care clinics in the area had been implementing Option B+ for at least 2 years, and our audit study indicated that implementation was closely aligned with the Malawian Ministry of Health guidelines.

**Figure 1.**
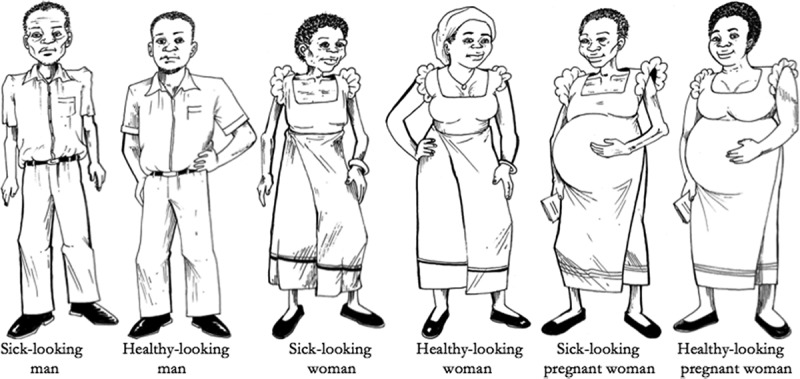
Cards used for card sort module.

In order to systematically investigate lay knowledge of and opinions about ART policy in Malawi, we implemented an interactive card sort exercise. During the course of the interview, interviewers presented respondents with six physical cards, each depicting a person with HIV (Figure 1). These six individuals were a (i) sick man; (ii) healthy-looking man; (iii) sick non-pregnant woman; (iv) healthy-looking non-pregnant woman; (v) sick pregnant woman; and (vi) healthy-looking pregnant woman. The interviewer then read the following instruction in Chichewa:

Not everyone who is HIV-positive can get ARVs right away. Since there are limited amounts, the Balaka clinics have to prioritize some people over others. There are six cards here that represent different people; each one of them has HIV. These six people are similar in every way except for the differences you can see: man-woman, healthy-unhealthy, pregnant-not pregnant.

Take a minute to look through the pictures on these six cards. I’d like you to think about the situation in the Balaka clinics right now and tell me whether you think this person would definitely get ARVs from the clinic now, maybe get ARVs from the clinic, or probably not get ARVs. Remember, all of these people have HIV. And you sort these into three piles for: Definitely, Maybe, and Probably not.

Next, respondents were asked to order the six cards, placing the person they thought most likely to get ART from the clinics first and the person who would receive them last on the other extreme with no ties allowed. Lastly, respondents were reminded that what actually happens in clinics is not always what one thinks *should* happen. Specifically, they were instructed:

Sometimes what actually happens in clinics isn’t what we think should happen. If it were up to you, how do you think ARVs should be distributed? Imagine a world where there are still shortages but you are the one deciding how to distribute ARVs in the most fair way. Who do you think should be the first to receive them and who should be last?

Although the level of abstraction in this set of tasks is high, TLT interviewers were thoroughly trained to introduce and explain the task, and TLT respondents had experience with card sort exercises from an earlier wave of the study [38]. Our analysis of the fieldnotes interviewers wrote after completing the section indicates that only five of 2,014 respondents had difficulty understanding the task.

We use these data to describe how young Malawians think ARVs *are* being distributed and how they think ARVs *should* be distributed. We present results separately for men and women. We then use sequence analysis [39,40] to depict the prevailing perceived and ideal prioritization sequences for ART distribution and optimal matching [40] to measure understanding of policy (operationalized as the proximity of perceived to actual policy sequences). We use chi-squared tests to assess gender differences and paired sample t-tests to measure distance between sequences. Lastly, we use multivariable ordered logistic regression to identify the correlates of awareness of ART prioritization policy including age, urban residence, education, HIV status/ART use, and an indicator of respondents’ (or for men, their main partners’) current pregnancy or recent birth (since January 2014). HIV status was based on HIV testing and counselling services offered at the end of the survey (94% acceptance), and ART use was self-reported.

## Ethics

Tsogolo la Thanzi was approved by the Social and Behavioral Sciences Institutional Review Board at The University of Chicago and Malawi’s National Health Sciences Research Committee. All study participants provided written informed consent.

## Results

Respondents’ characteristics are described in Table 1. Female respondents were between the ages of 21–31 in 2015, and their male partners were, on average, five years older. 14.8% of women and 9.2% of men were HIV positive, similar to national estimates (NSO and ICF Macro 2011). More than half of HIV-positive women were on ART, while just over one-third of HIV-positive men were receiving treatment.

**Table 1.** Sample description, TLT-2015

	Women	Men (partners)
Age, mean (SD)	25.6 (3.3)	31.0 (6.1)
Years of education, mean (SD)	8.0 (3.1)	7.4 (3.4)
Lives near a trading centre, %	37.1	29.4
Currently married, %	72.1	92.5
HIV category, %		
HIV negative/unknown	85.2	90.8
HIV+ not on ART	6.5	5.9
HIV+ on ART	8.3	3.3
Recent birth or current pregnancy, %	36.7	
Partner had recent birth or current pregnancy, %		44.8
N	1440	574

**Figure 2.**
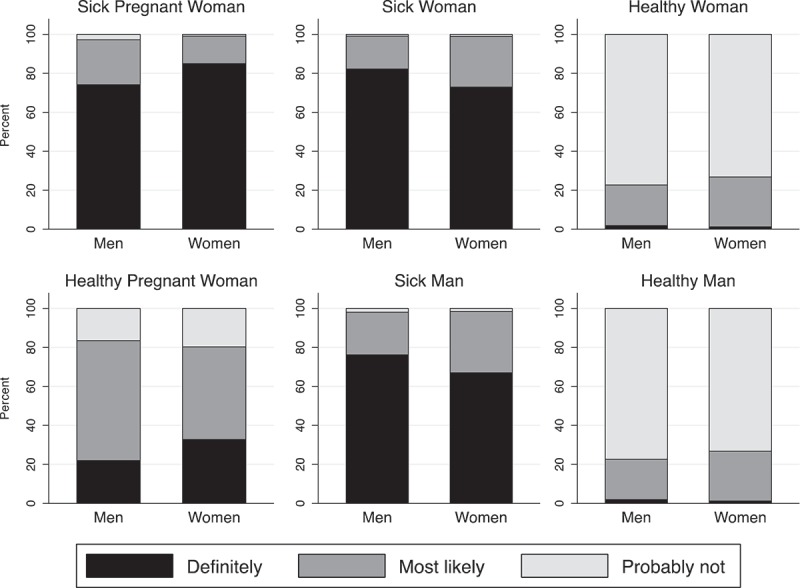
Perceptions of ART policy, TLT 2015.

Figure 2 depicts lay perceptions of who gets ART by gender. As a reminder, respondents were asked whether the person represented on each of the six cards would *definitely* receive ART (black), *maybe* receive ART (dark grey), or *probably not* receive ART (light grey) at local clinics. The unfortunate reality that supplies are insufficient to meet the needs of the entire infected population is acutely perceived at the population level: only one respondent believes that all six people represented by the cards would *definitely* receive ART and only 7.9% that all sick and pregnant people would *definitely* receive. Five notable patterns stand out in Figure 2: (1) there is general consensus that sick individuals receive ART; (2) more men than women believe sick men (76.1% vs. 67.0%; p <0.001) and women (82.2% vs. 73.0%; p <0.001) will definitely get ART; (3) more women than men believe sick pregnant women will definitely get ART (84.9% vs. 74.3%, p <0.001); (4) very few respondents imagine that healthy women or men can access ART; and (5) there is no consensus about what happens to healthy-pregnant women: 47.4% of women and 61.5% of men see pregnant women as possible recipients of ART (i.e. maybe).

**Figure 3.**
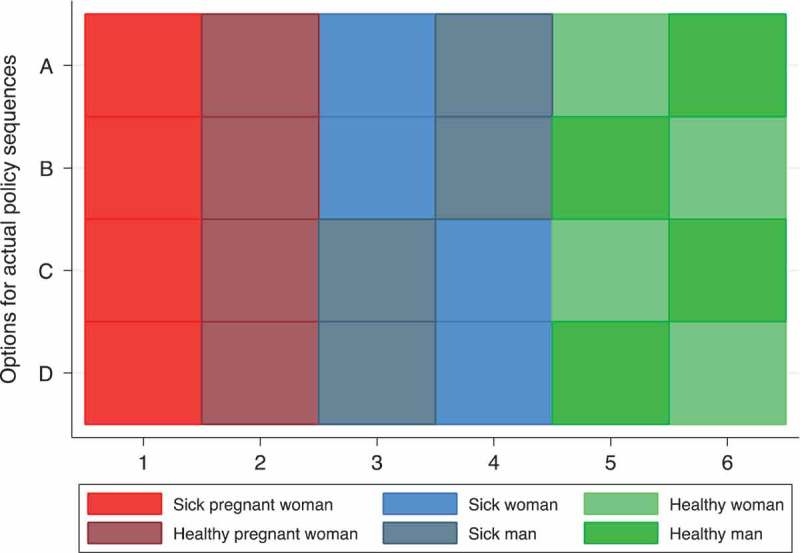
Four depictions of actual policy sequences for ART prioritization under Option B +.

We shift to a sequence framework for visualizing local understandings and evaluations of ART availability as a set of ordered priorities. Malawi’s ART policy under Option B+ was fixed in terms of its guiding priorities (pregnancy and medical eligibility) but ambiguous about gender, given equivalent health status. The distribution strategy could be represented by any one of the four sequences displayed in Figure 3, wherein “1” on the x-axis indicates first priority. Theoretically, men and women who met medical eligibility criteria and all pregnant women were *eligible* for ART. In practice, however, health clinics prioritized pregnant women over those eligible through CD4 count or WHO staging. The manuals for healthcare workers emphasized that HIV+ pregnant women start ART *the same day* they are diagnosed, while eligible others initiate *within 7 days* and *must* attend group counselling first [41]. Pregnant and breastfeeding women were referred to as a special group, “‘universally eligible’ because they have been prioritized for immediate and lifelong ART” [42].

**Figure 4.**
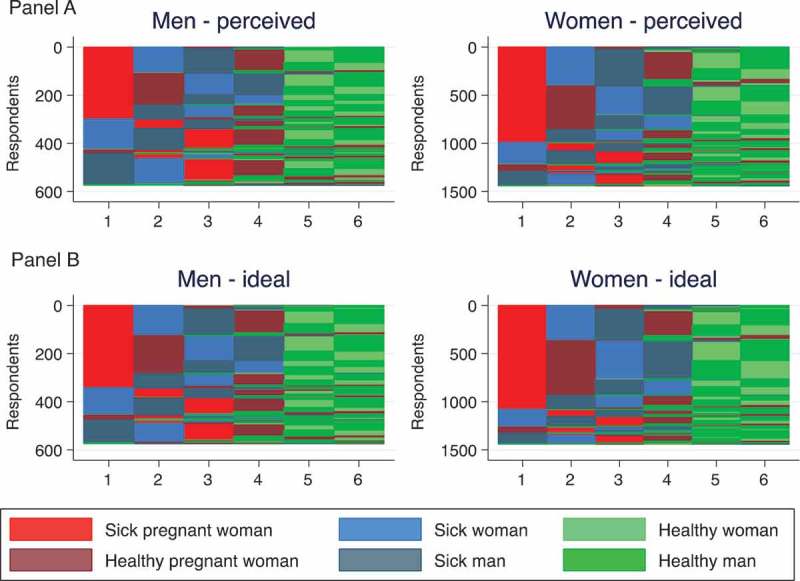
Sequence plots of perceived ART allocation priorities and ideal priorities by gender.

Figure 4 presents sequences illustrating how young adults in Balaka understand the logic of ART distribution (Panel A) and what they think constitutes an ideal policy (Panel B). Only 30.7% of women and 22.1% of men accurately assessed the policy, meaning that their sequence matched one of the four depicted in Figure 3. Another 42.3% of women and 31.2% of men described a prioritization sequence that was just one step away from policy. The most common difference was positioning healthy-pregnant women behind sick men and women; in other words, the most common misconception was precisely the change Option B+ introduced.

**Figure 5.**
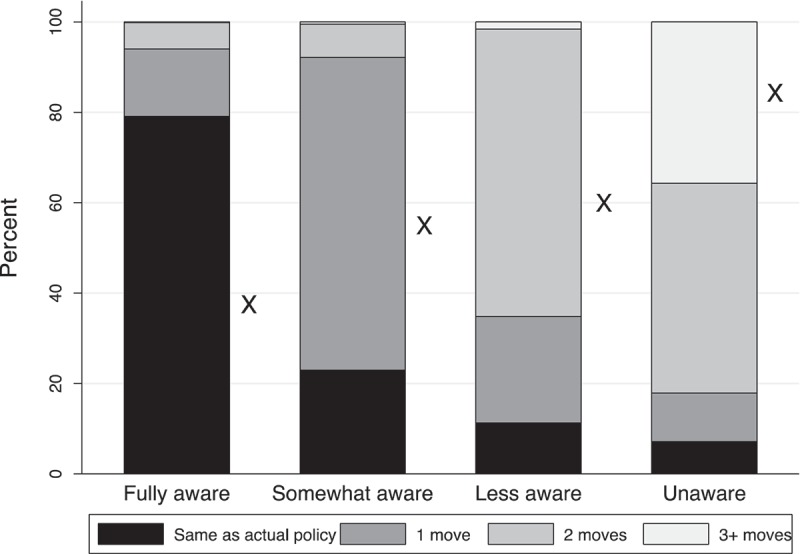
Alignment of ideal and actual policy sequence by policy awareness.

When asked to reorder the cards to reflect what they thought to be the *fairest* approach to distributing ART, about half of respondents moved cards. The Xs in Figure 5 show that the likelihood of having to move cards to articulate ideal policy is negatively related to the respondent’s level of policy awareness (x-axis). Categories within the stacked bars refer to the proximity of ideal sequences to the closest actual policy sequence (measured as number of moves required to convert one sequence to another). Respondents with high levels of policy awareness articulate ideals that align closely with actual policy, but this is not true of those with lower levels of awareness (denoted by the bars in Figure 5). Note that because there are four possible sequences representing actual policy, it is possible for respondents to report a perceived sequence that aligns with actual policy, move a card, and still have an ideal sequence consistent with actual policy.

Interestingly the policy ideals of young adults in Malawi are more closely aligned with actual policy than with their perceptions of how ART is allocated (p <0.001 for both sexes). The modal ideal sequence (identical for both sexes) is: (1) sick pregnant woman, (2) healthy-pregnant woman, (3) sick woman, (4) sick man, (5) healthy woman, (6) healthy man. The simplest analytical lever on the perceived fairness of Option B+ is the placement of the healthy-pregnant woman card between their perceived-allocation sequence (Figure 4, Panel A) and their ideal sequence (Figure 4, Panel B). Both men and women were more likely to move healthy-pregnant women forward than to demote them (19.7% vs. 11.0% for men; 21.7% vs. 11.1% for women; p <0.001 for both). Overall, 26.5% of men and 36.8% of women believe the policy prioritizes healthy-pregnant women over sick men and women, but even greater percentages (31.9% and 44.5%, respectively) think this is how ART should be allocated (p <0.001 for both sexes).

**Figure 6.**
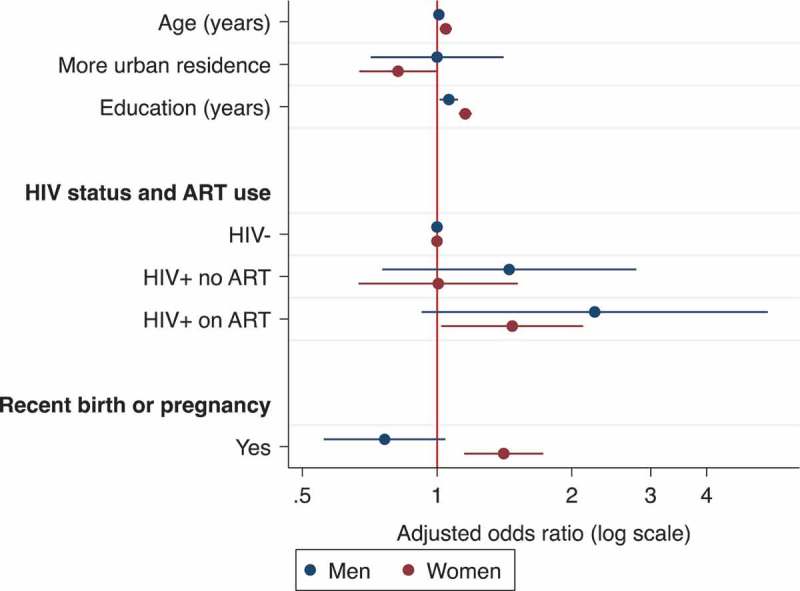
Association between awareness of ART prioritization policy and various factors. Adjusted odds ratios estimated from multivariable ordered logistic regression of proximity between perceived and actual policy by gender.

Figure 6 presents odds ratios from multivariable ordered logistic regression models that establish how awareness of ART policy prioritization under Option B+ is patterned. More accurate understandings are associated with education for men, and with age, education and more rural residence for women. Certain health experiences engender connection to health facilities, thereby enhancing knowledge of policy. Being HIV positive is only associated with more accurate knowledge of policy if the respondent is on ART (women: aOR 1.47, 95% CI 1.02–2.12, *p = *0.038; trending towards a positive relationship for men: aOR 2.24, 95% CI 0.92–5.48, *p = *0.074). Net of HIV status, a recent pregnancy (proxy for exposure to antenatal services) is positively associated with awareness for women (aOR 1.41, 95% CI 1.15–1.73, *p = *0.001) but not men (aOR 0.76, 95% CI 0.56–1.04, *p = *0.091). This is likely due to the fact that although men are officially encouraged to attend antenatal care with their partner, few do [43,44].

## Discussion

We sought to assess awareness and perceived fairness of the ART prioritization system under Option B+ in Malawi from the perspective of those most affected by changes to the prioritization system. We found that although the vast majority of our sample was aware that the sick were prioritized over healthy HIV-positive individuals, there was confusion about treatment prospects for healthy-pregnant women. Four years into Option B+, less than a third of women and a quarter of men understood how ART was being distributed under the policy. This lack of awareness reflects the way the policy was rolled out. Although the rationale for the system was articulated to international audiences and to practitioners within Malawi [13,14], in contrast to voluntary medical male circumcision (VMMC) and many other new health initiatives [45], Option B+ was introduced without any public outreach campaign.

Our study is subject to limitations. While our use of illustrations clearly conveyed the distinction between “healthy” and “sick” populations, it does not translate precisely to the medical eligibility criteria employed in health centres. Additionally, because the men in the sample were enrolled through their randomly selected female partners, we have fewer men and they do not constitute a representative sample. Finally, the card-sort approach lacks the nuance afforded by in-depth qualitative analyses but does provide what we believe to be the first population-perspective on what young adults know and think about Option B +.

More broadly, our findings indicate the prevailing avenues by which ordinary people learn about ART policies. Our results suggest that Malawians become aware of ART policy through their engagement with the ART allocation process itself – either as ART recipients or through antenatal care. Unfortunately, those most affected by the reprioritization – the HIV positive not yet on ART – have no such advantage.

Insufficient transparency is not merely an academic concern; it threatens the moral legitimacy of policies and policymakers [31] and can impede any programme’s success. Challenges with uptake and default among HIV-positive pregnant women under Option B+ [46–49] might be partially explained by women’s confusion at being told to start ART the same day they are tested and stay on it regardless of how they feel, when they know symptomatic people – sometimes their own husbands – who do not yet have access [50–52]. If women, and the men who support them, understood the policy and the reasons behind it, they might be better ART users. Each new policy – provider-initiated HIV testing, VMMC, and universal test-and-treat – provides an opportunity to enhance or compromise levels of trust in the medical establishment. Where mistrust is already present, as it is in Malawi and across much of SSA [53–57], confusion invites rumours and conspiracy theories that further erode the possibility of success [58].

Our evidence suggests that despite limited community consultation the prioritization of pregnant women made explicit by Option B+ resonates with the sensibilities of most TLT respondents. A sizeable majority believes that sick pregnant women should be the first to receive ART, and the belief that healthy-pregnant women should be favoured over sick populations is more prevalent than is the knowledge that this was being done over the previous four years. Importantly, Malawians who understood the ART policy were more likely to view it as fair.

Contrary to our expectation that men might view pregnant women’s accelerated access to ART as unfair, we find no evidence that this is true. Even so, it is important to monitor how new ART policies affect existing gender disparities in ART access. Recent evidence from Malawi shows that Option B+ substantially increased pregnant women initiating ART without reducing the number of new initiates among men or non-pregnant women [59]. This shift has, however, reduced the *proportion* of male initiates [59] and may exacerbate existing disparities wherein men are less likely to be on ART, initiate later, and are more likely to die of AIDS [23,60].

We focus here on applying the principles of transparency and relevance to the case of Option B+ in Malawi [31,34]. In contexts of scarcity, systems to allocate limited resources are both necessary and necessarily contested. They should, however, be communicated to and understood by the populations they affect [4].

As Malawi and neighbouring countries shift to models of universal test-and-treat, policymakers would do well to keep the principles of transparency and relevance at the fore. Even as treatment access expands and improves, the reigning understanding in Malawi is that not all HIV-positive people can receive ARVs. Our view is that it would be unrealistic to expect improvements in retention in the absence of clear communication about what ART policy is and without providing the wider population with basic explanations for on-going shifts in policy.

## Conclusions

Young adults in southern Malawi had limited awareness of the ART prioritization system under Option B +. Nonetheless, many people support prioritizing otherwise healthy HIV-positive pregnant women above the sickest populations. As policymakers rollout new ART policies and continue to restructure access to these lifesaving medicines, they have a responsibility to communicate the policies and their rationales to local communities whose access is being negotiated. Indeed doing so would not only be more ethical but would likely improve the policies’ odds of success.
